# *NCOA6* knockdown enhances RSL3-induced ferroptosis in pancreatic cancer cells and increases the sensitivity to gemcitabine: *NCOA6* knockdown and RSL3-induced ferroptosis in pancreatic cancer

**DOI:** 10.3724/abbs.2024221

**Published:** 2025-03-10

**Authors:** Yuming Jia, Zeng Ye, Xin Wang, Yanli Deng, Chao Wang, Zhilei Zhang, Guixiong Fan, Wuhan Yang, Xiaowu Xu, Yi Qin, Li Peng

**Affiliations:** 1 Department of Hepatobiliary Surgery the Fourth Hospital of Hebei Medical University Shijiazhuang 050000 China; 2 Department of Emergency the Fourth Hospital of Hebei Medical University Shijiazhuang 050000 China; 3 Department of Clinical Laboratory the Fourth Hospital of Hebei Medical University Shijiazhuang 050000 China; 4 Department of Pancreatic and Hepatobiliary Surgery Fudan University Shanghai Cancer Center Shanghai 200031 China; 5 Pancreatic Cancer Institute Fudan University Shanghai 200031 China; 6 Hebei Province Key Laboratory of Precision Diagnosis and Therapy for Hepatobiliary-Pancreatic Malignancies the Fourth Hospital of Hebei Medical University Shijiazhuang 050000 China

**Keywords:** NCOA6, pancreatic cancer, ferroptosis, gemcitabine

## Abstract

Ferroptosis is a type of programmed death characterized by iron-dependent lipid peroxidation, and targeting ferroptosis has been shown to efficiently kill highly aggressive cancer cells. Previously, we confirmed that nuclear receptors regulate ferroptosis in pancreatic cancer. However, whether nuclear receptor co-activators regulate ferroptosis is unclear. Here, we show that knocking down the nuclear receptor co-activator, *NCOA6*, enhances the sensitivity of pancreatic cancer cells to ferroptosis. Mechanistically, *NCOA6* knockdown promotes the expression of ACSL4 while inhibiting the expression of SCD1, resulting in changes in lipid metabolism, sensitivity to RSL3-induced ferroptosis, and sensitivity to gemcitabine in pancreatic cancer. The relationships between NCOA6 and ACSL4 or SCD1 are further explored in clinical specimens. This study reveals that targeting NCOA6 might alleviate gemcitabine resistance in pancreatic cancer.

## Introduction

Pancreatic cancer is a malignant tumor that seriously threatens human health. The overall 5-year survival rate is only approximately 6%. At present, the main treatment method for cancer is still surgery-based comprehensive treatment, but the surgical resection rate of this disease is low, the effect is poor, and chemotherapy is the main method of systemic treatment. Gemcitabine is a commonly used chemotherapeutic drug for pancreatic cancer that can induce the apoptosis of pancreatic cancer cells [1]. However, the resistance of pancreatic cancer to gemcitabine still affects the effectiveness of chemotherapy, which is an urgent problem [2].

Ferroptosis is a programmed death mode different from cell apoptosis, with the core event being the production of reactive oxygen species (ROS) and subsequent lipid peroxidation mediated by hydroxyl radicals (-OH), leading to membrane damage. Ferroptosis is regulated by multiple signaling or metabolic pathways. Erastin and RSL3 are ferroptosis inducers that selectively kill cancer cells with RAS mutations [ 3, 4]. However, KRAS gene mutations are common in pancreatic cancer; therefore, pancreatic cancer is likely to be potentially sensitive to ferroptosis. The study of the relationship between pancreatic cancer and ferroptosis may have potential value for the treatment of pancreatic cancer.

NCOA6 is a nuclear receptor coactivator that participates in cellular transcription, survival, growth, and development [ 5, 6]. It is also a gene that promotes anti-apoptosis and survival. This gene is highly expressed in various cancers, including breast cancer, lung cancer, pancreatic cancer, melanoma, colorectal cancer, and other tumors [ 5, 7– 11]. Overexpression of the *NCOA6* gene is associated with poor prognosis in multiple malignancies, and studies have shown that it can promote cell invasion and migration by activating NF-κB [12]. For example, in hepatocellular carcinoma (HCC), its knockdown can disrupt cell proliferation, migration, and invasion [13]. At present, few reports exist on the role of this gene in ferroptosis. However, as an important transcription factor involved in various biological behaviors of cells, its regulatory role is extensive, and the process of ferroptosis is regulated by multiple pathways. Therefore, we believe that it is highly likely to participate in the process of ferroptosis.

In the present study, we found that knockdown of the *NCOA6* gene can increase the sensitivity of pancreatic cancer cells to ferroptosis. Knockdown of *NCOA6* promotes the expression of acyl coenzyme a synthase long chain family member 4 (ACSL4) while inhibiting the expression of stearoyl coenzyme a desaturase 1 (SCD1), resulting in changes in lipid metabolism and sensitivity to RSL3-induced ferroptosis in pancreatic cancer cells. The sensitivity to gemcitabine was increased in pancreatic cancer cells with *NCOA6* knockdown.

## Materials and Methods

### Cell culture

The human pancreatic cancer cell lines PANC-1 and SW1990 were obtained from the American Typical Culture Collection (ATCC; Manassas, USA). PANC-1 cells were cultured in Dulbecco’s modified Eagle’s medium (DMEM; HyClone, Carlsbad, USA) supplemented with 10% fetal bovine serum (FBS; Wisent, Saint-Jean-Baptiste, Canada), and SW1990 cells were cultured in L-15 medium (Corning, New York, USA) supplemented with 10% FBS. All media contained 100 U/mL penicillin and 100 μg/mL streptomycin (Yuanpei, Shanghai, China). The cells were cultured in a 37°C incubator containing 5% CO _2_.

### Plasmids and shRNAs

The pLKO.1-TRC cloning vector (Addgene Plasmid 10878; SBI, Palo Alto, USA) was used to generate shRNA constructs to downregulate NCOA6 expression. pLKO.1-sh-scramble (Addgene plasmid 1864) was used as a control plasmid. The lentivirus was produced by cotransfecting the *NCOA6* silencing construct with psPAX2 and pMD2. G vectors into HEK293T cells at a ratio of 4:3:1. Stable *NCOA6*-silenced cell lines were generated via infection of PANC-1 and SW1990 cells and subsequent selection with puromycin. The encoding sequence is as follows: NCOA6-sh-1: 5′-GCAGATTATGACCAAATCAAAT-3′; and NCOA6-sh-2: 5′-ACAAATGAACCCAGCTAATTT-3′.

### Cell treatment

For drug treatment, cells were seeded into 6-well plates (1 × 10 ^6^ cells/well) and allowed to adhere for 24 h. Then, based on the grouping, the cells were exposed to the corresponding treatment drugs dissolved in DMSO for 24 h. Control groups received DMSO vehicle. After treatment, cells were harvested using 0.25% trypsin-EDTA and washed twice with PBS for subsequent assays. All experiments were performed in triplicate.

### Cell viability assay

A Cell Counting Kit-8 (CCK-8; Dojindo Laboratory, Tokyo, Japan) was used to detect cell viability according to the manufacturer’s instructions. In brief, first, a cell suspension with 1000 cells per well was inoculated onto a 96-well plate, and then the plate was incubated in a culture incubator for 24 h. The test drugs at different concentrations (0–30 μM) were added to the wells of a 96-well plate, which was then placed in an incubator for another 72 h for cultivation. Then, 10 μL of CCK8 solution was added to each well. The plate was incubated in the incubator for another 4 h. The absorbance at 450 nm was measured via a spectrophotometer (BioTek, Winooski, USA).

### RNA extraction and quantitative PCR

In brief, total RNA was extracted via an Ultra Pure RNA kit (CWBIO, Shanghai, China). cDNA was obtained through reverse transcription via the TaKaRa PrimeScript RT kit (TaKaRa, Dalian, China). The QuantStudio 6 Flex real-time PCR system (Thermo Fisher Scientific, Waltham, USA) was used to determine the expression of the target genes and *β-actin*. We used the 2 ^–ΔΔCT^ method to quantify the relative expression of each target gene three times. The sequences of primers used are shown in Table 1.

**Table:** **Table 1** Sequences of the primers used in this study

Gene	Primer sequence (5′→3′)
*NCOA6*	Forward: ACCGTTGCCTGGAGAACAAGGA
	Reverse: GAGTTGAGGAGGCATCTGCTGA
*GPX4*	Forward: ACAAGAACGGCTGCGTGGTGAA
	Reverse: GCCACACACTTGTGGAGCTAGA
*SLC7A11*	Forward: TCCTGCTTTGGCTCCATGAACG
	Reverse: AGAGGAGTGTGCTTGCGGACAT
*SCD1*	Forward: CCTGGTTTCACTTGGAGCTGTG
	Reverse: TGTGGTGAAGTTGATGTGCCAGC
*ACSL4*	Forward: GCTATCTCCTCAGACACACCGA
	Reverse: AGGTGCTCCAACTCTGCCAGTA
*NRF2*	Forward: CACATCCAGTCAGAAACCAGTGG
	Reverse: GGAATGTCTGCGCCAAAAGCTG
*RRM1*	Forward: AAAGGAAGAGCAGCGTGCCAGA
	Reverse: ACCTCATCCAGACCAGGACACT
*dCK*	Forward: AGTGGTTCCTGAACCTGTTGCC
	Reverse: GACCATCGTTCAGGTTTCTCATAC
*hENT*	Forward: GAGCAGGCAAAGAGGAATCTGG
	Reverse: ACGGCTGGAAACATCCCAATGG
*β-actin*	Forward: TCCTTCCTGGGCATGGAGT
	Reverse: CAGGAGGAGCAATGATCTTGAT

### MDA experiment

In brief, a Lipid Peroxide Malondialdehyde Detection kit (S0131M; Beyotime, Shanghai, China) was used to measure the production of MDA in pancreatic cancer cells. The cells were collected and subjected to protein quantification via a BCA assay kit (Epizyme, Cambridge, USA) and MDA detection according to the manufacturer’s protocols.

### Lipid peroxidation experiment

A BODIPY™ 581/591 C11 fluorescence probe (D3861; Thermo Fisher Scientific) was used to perform the lipid peroxidation experiment. In brief, the cells were treated with a specified drug for a specified time period, digested with trypsin, resuspended in 400 μL of medium containing BODIPY™581/591 C11 (2 μM), and incubated at 37°C in a cell incubator for 30 min. Then, the samples were analyzed by flow cytometry, and data were collected from the FL1 channel. The number of cells analyzed under each condition should not be less than 10000. In addition, the adherent cells were incubated directly with BODIPY™581/591 C11 (2 μM) and observed under a fluorescence microscope (Nikon, Tokyo, Japan).

### Western blot (WB) analysis

Proteins were extracted using RIPA lysis buffer (Beyotime) containing protease inhibitors (Beyotime). The protein concentration was determined via an Omni Easy™ Instant BCA protein detection kit (Epizyme). Equal amounts of total protein were separated via SDS-PAGE and transferred to PVDF membranes (Millipore, Billerica, USA). The membranes were blocked with protein-free rapid blocking buffer (Epizyme) at room temperature for 15 min, after which the membranes was incubated with primary antibodies including: rabbit anti-human NCOA6 antibodies (DF13176, 1:1000; Affinity, Cincinnati, USA) or rabbit anti-human GPX4 (A11243), NRF2 (A1244), SCD1 (A16429), SLC7A11 (A25291), ACSL4 (A6826), hENT (A13204), RRM1 (A4259), dCK (A1794), and β-actin (AC028) antibodies (ABclonal, Wuhan, China), followed by incubation with HRP-conjugated sheep anti-rabbit IgG secondary antibody (ABclonal). The target proteins were visualized via an enhanced chemiluminescence (ECL) plus western blotting detection system (Tanon, Shanghai, China).

### Co-immunoprecipitation (IP)

After being washed with pre-cooled PBS and lysing with cold WB and IP lysis buffer (containing 1 mM PMSF), the PANC-1 and SW1990 cells were scraped to clean 1.5-mL Eppendorf tubes and centrifuged at 14,000 *g* at 4°C for 10 min. The supernatant was quantified via a BCA assay kit and prepurified with rabbit IgG (Beyotime) and pre-treated with protein A/G magnetic beads (Beyotime). Then, the samples were incubated with an appropriate amount of anti-NCOA6 antibody (DF13176; Affinity) and slowly shaken overnight on a rotating shaker at 4°C. The next day, the samples were incubated with pre-treated protein A/G magnetic beads for 3 h. The sediment was collected, washed with PBS, and mixed with 2× SDS protein loading buffer for western blot analysis using rabbit anti-human antibody SREBP1(14088-1-AP; Proteintech, Wuhan, China).

### Immunohistochemical (IHC) staining

The clinical tissue samples were obtained from patients diagnosed with pancreatic cancer at Fudan University Shanghai Cancer Center, with patient consent and approval from the Institutional Research Ethics Committee. Antibodies against SCD1, ACSL4 and NCOA6 were used to conduct immunohistochemical staining in paraffin-embedded tissues according to standard IHC procedures. Anti-ACSL4 antibody (A6826; ABclonal), anti-SCD1 antibody (ab23686868; Abcam, Cambridge, UK) and anti-NCOA6 antibody (DF13176; Affinity) were used at a dilution of 1:100. Positive proportions and intensities were semi-quantitatively scored as previously described [14].

### Statistical analysis

All the statistical analyses were performed via IBM SPSS 26 or GraphPad Prism 8. Quantitative data are presented as the mean ± SD, an independent sample *t* test was used to compare two sets of normally distributed quantitative data, and Spearman correlation analysis was used to analyze the correlation between grade data. Statistical significance was considered when the *P* value was less than 0.05.

## Results

### *NCOA6* knockdown enhances RSL3-induced ferroptosis

**Figure 1:**
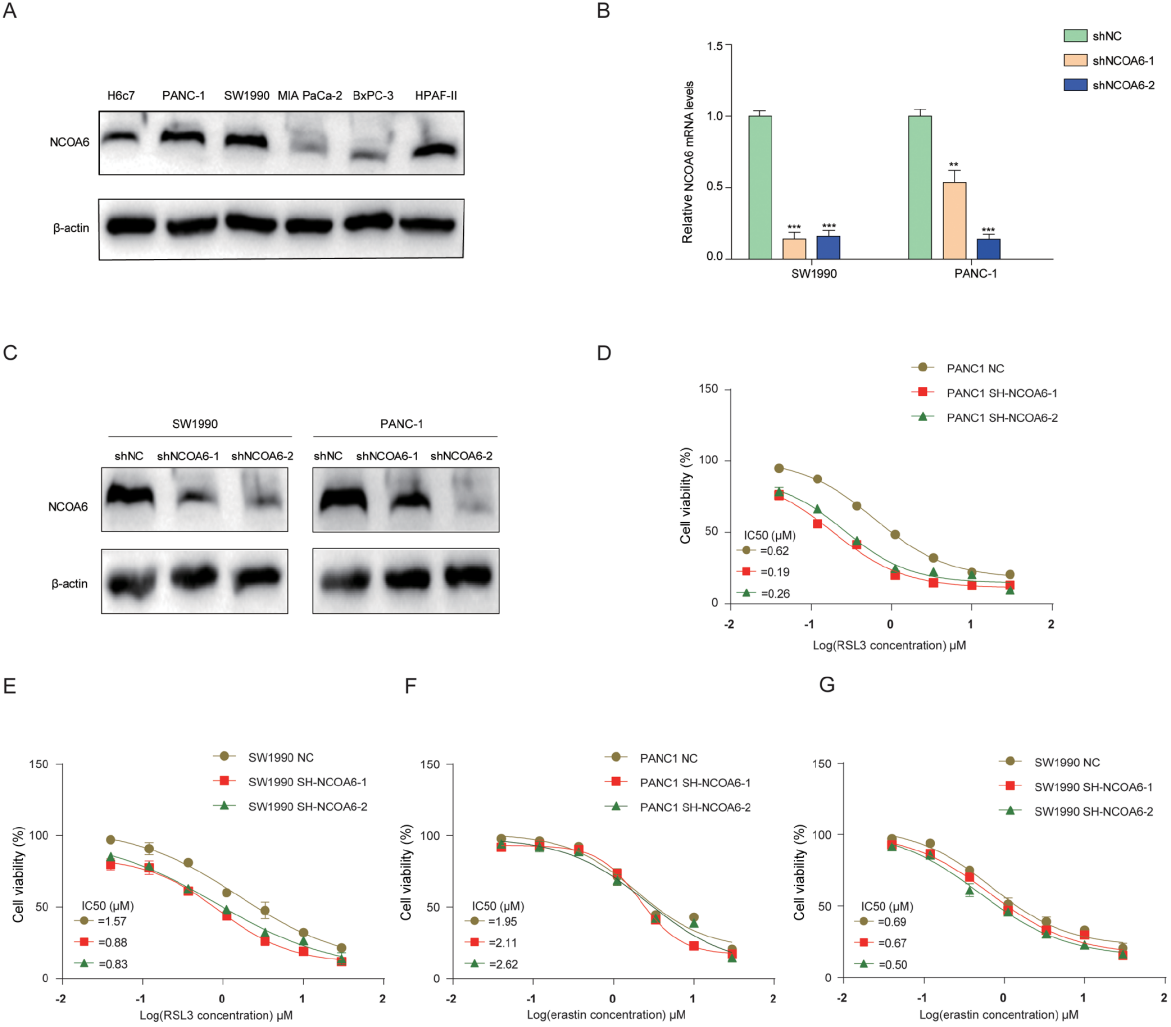
Figure 1 *NCOA6* knockdown enhances RSL3-induced ferroptosis (A) Western blot analysis of the protein expression of NCOA6 in different cell lines. (B) The knockdown effect of sh-NCOA6 was analyzed by qRT-PCR. (C) Western blot analysis of the effect of sh-NCOA6 knockdown. (D–G) Dose-dependent toxic effects of RSL3 and erastin in sh-NCOA6 or sh-RNA-NC stable SW1990 and PANC-1 cell lines are shown, and the IC50 values were calculated. **P < 0.01, ***P < 0.001.

We detected the protein expression level of NCOA6 in a pancreatic duct epithelial cell line (H6C7) and several pancreatic cancer cell lines. The results revealed that the basic expression of NCOA6 was relatively high in the SW1990 and PANC-1 cell lines ( Figure 1A), so we chose these two cell lines for subsequent experiments. Next, we generated stable sh-RNAs via these two cell lines and further validated the knockdown efficiency through quantitative RT-PCR and western blot analysis ( Figure 1B,C). To test whether *NCOA6* gene knockdown can regulate ferroptosis in pancreatic cancer cells, we tested the sensitivity of two cell lines to two typical ferroptosis inducers, RSL3 and erastin. Cells were treated with different concentrations (0–30 μM) of RSL3 or erastin, and then cell viabilities were measured. The results revealed that cells with *NCoA6* knockdown demonstrated significantly enhanced sensitivity to RSL3-induced ferroptosis, while showing no significant enhancement in response to erastin-induced ferroptosis ( Figure 1D–G). These results suggested that NCOA6 regulated RSL3-induced ferroptosis in pancreatic cancer cells.

### *NCOA6* knockdown does not lead to lipid peroxidation but enhances RSL3-induced lipid peroxidation

**Figure 2:**
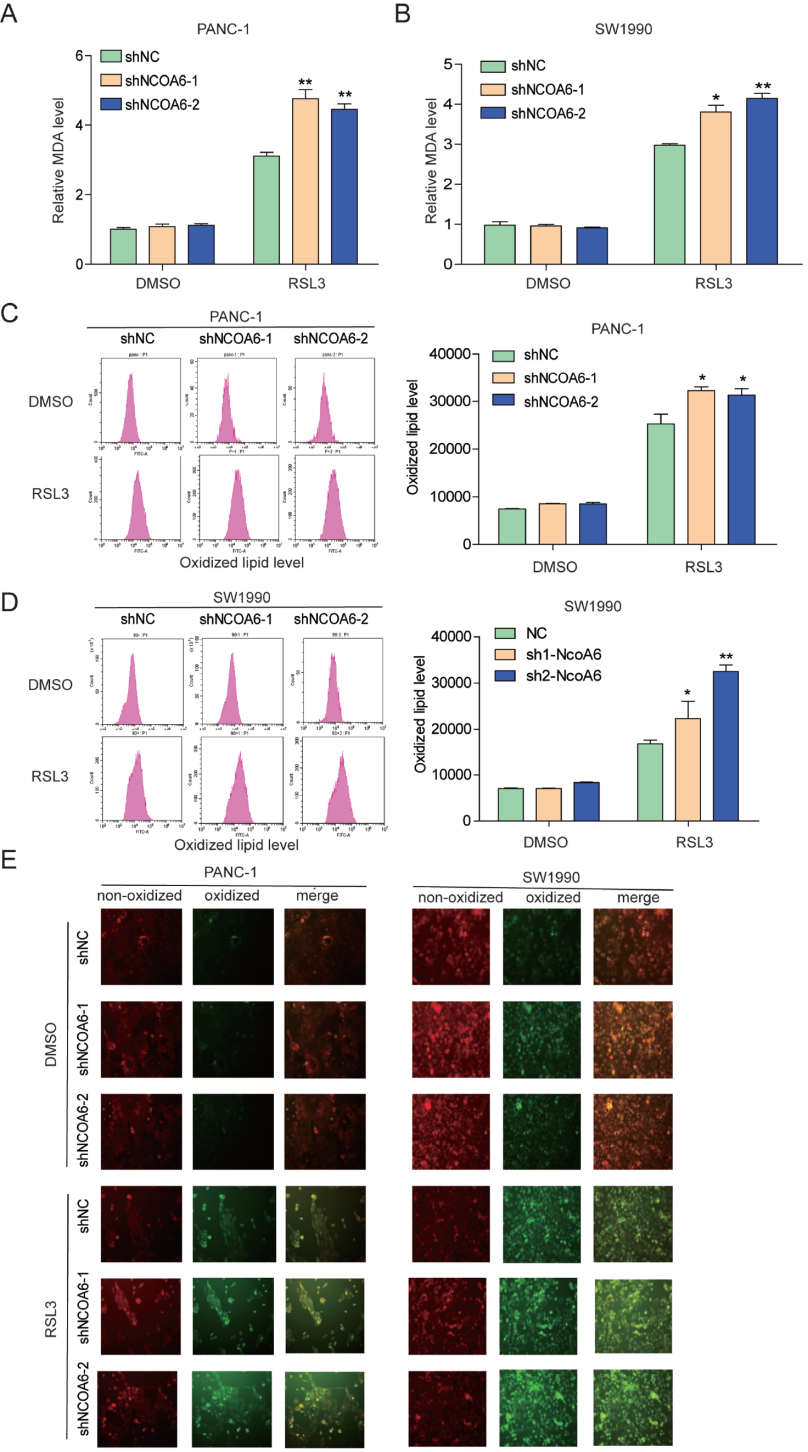
Figure 2 *NCOA6* knockdown does not lead to lipid peroxidation but enhances RSL3-induced lipid peroxidation (A,B) MDA levels were detected in NCOA6-knockdown SW1990 and PANC-1 cell lines treated with or without RSL3. (C,D) Flow cytometry was used to detect oxidative lipids in NCOA6-knockdown SW1990 and PANC-1 cells treated with or without RSL3. (E) Observation of non-oxidized (red) and oxidized (green) lipids in NCOA6-knockdown SW1990 and PANC-1 cell lines with or without RSL3 treatment via fluorescence microscopy (Maginfication, 100×). *P < 0.05, **P < 0.01.

Lipid peroxidation is an important feature of ferroptosis. MDA is a product of lipid peroxidation, and its content reflects the level of lipid peroxidation. Our study revealed that after the *NCOA6* gene was knocked down, the MDA levels in SW1990 and PANC-1 cells were not affected, but when the cells were stimulated with RSL3 (1 μM), the MDA levels significantly increased ( Figure 2A,B). Next, we directly detected oxidized lipids using the fluorescence probe BODIPYTM 581/591 C11. When the probe binds to oxidized lipids, the fluorescence changes from red to green. We analyzed fluorescence by flow cytometry and found that *NCOA6* gene knockout did not affect lipid oxidation levels but promoted RSL3-induced lipid oxidation ( Figure 2C,D). Further verification was then conducted via fluorescence microscopy, and RSL3 resulted in increased lipid oxidation in *NCOA6*-knockdown cells ( Figure 2E). These results indicated that NCOA6 enhanced RSL3-induced lipid peroxidation in pancreatic cancer cells.

### *NCOA6* gene regulates sensitivity to RSL3 through SCD1 and ACSL4

**Figure 3:**
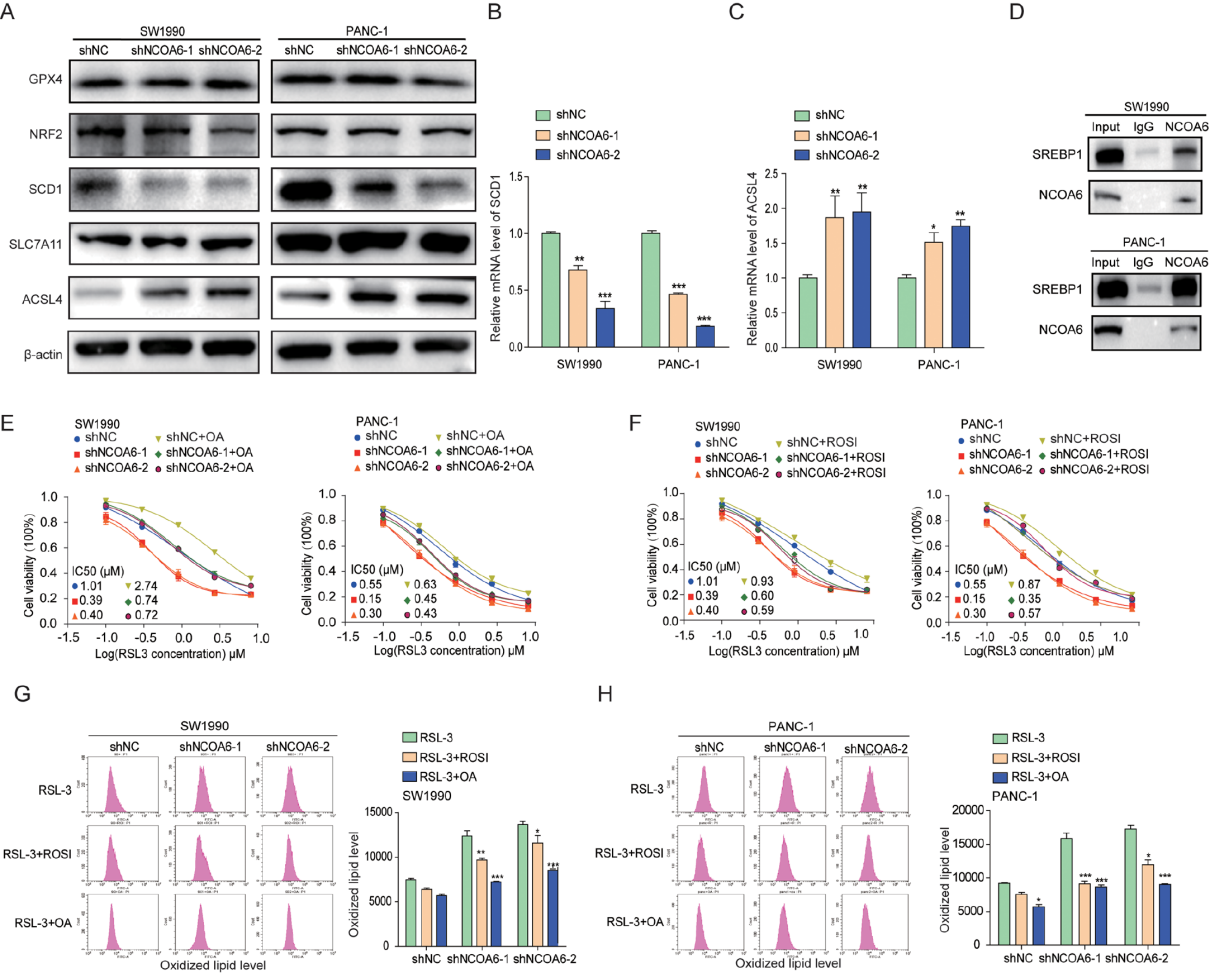
Figure 3 *NCOA6* gene regulates sensitivity to RSL3 through SCD1 and ACSL4 (A) Western blot analysis of the protein expressions of GPX4, NRF2, SCD1, SLC7A11 and ACSL4 in the pancreatic cancer cell lines SW1990 and PANC-1 with and without NCOA6 knockdown. (B,C) qRT-PCR analysis revealed changes in the mRNA levels of ACSL4 and SCD1 after NCOA6 was knocked down in SW1990 and PAC-1 cell lines. (D) Co-IP was used to analyze the interaction between SREBP1 and NCOA6. (E,F) Dose-dependent toxicity of RSL-3 on NCOA6-knockdown SW1990 and PANC-1 cells after ROSI or OA pre-treatment. (G,H) Flow cytometry was used to detect lipid oxidation levels induced by RSL3 in SW1990 and PANC-1 cell lines with and without NCOA6 knockdown under ROSI and OA pretreatment. *P < 0.05, **P < 0.01, ***P < 0.001.

RSL3 mediates ferroptosis through direct action on GPX4. Our results showed that knocking down *NCOA6* increased the degree of lipid oxidation induced by RSL3. However, through western blot analysis experiments, we found that knocking down the *NCOA6* gene did not affect the expression of GPX4. We also detected the expressions of NRF2, SCD1, SLC7A11, and ACSL4. ACSL4 participates in the biosynthesis and remodeling of phosphatidylethanolamine (PE), activates polyunsaturated fatty acids, and affects the transmembrane properties of polyunsaturated fatty acids. The expression of ACSL4 enhances the sensitivity to ferroptosis compounds [15]. SCD1 can promote synergistic effects with ACSL4, increasing sensitivity to ferroptosis [16]. Our results revealed that after *NCOA6* knockdown, SCD1 was downregulated, whereas ACSL4 was upregulated ( Figure 3A). Moreover, qRT-PCR confirmed that the mRNA levels of SCD1 and ACSL4 were also altered ( Figure 3B,C). SREBP1 is regulated by ACSL4 [17] and can regulate downstream SCD1 [18]. Therefore, we tested whether there is an interaction between NCOA6 and SREBP1, further confirming the regulatory effect of NCOA6 on SCD1 and ACSL4. Co-IP experiments revealed an interaction between NCOA6 and SREBP1 ( Figure 3D). Next, we validated whether *NCOA6* gene knockdown increases cell sensitivity to RSL3 by regulating the expressions of SCD1 and ACSL4.

SCD1 has been reported to promote the production of monounsaturated fatty acids that are resistant to ferroptosis and can inhibit the sensitivity of cells to RSL3-induced ferroptosis [ 16, 19]. Oleic acid (OA) is one of the end products of SCD1, and we found that OA (500 μm) partially restored the viability of *NCOA6*-knockdown cells treated with RSL3. Therefore, NCOA6 regulated the sensitivity of cells to RSL3-induced ferroptosis by affecting the expression of SCD1 ( Figure 3E).

ACSL4 was upregulated in *NCOA6*-knockdown pancreatic cancer cells. ACSL4 can promote lipid peroxidation [17]. To further verify whether *NCOA6* knockdown increases the RSL3 sensitivity of pancreatic cancer cells through the upregulation of ACSL4 expression, we treated pancreatic cancer cells with rosiglitazone (20 μm), a pharmacological inhibitor of ACSL4, and found that it restored the viability of the RSL3-treated cells to a certain extent ( Figure 3F).

Next, we used the BODIPYTM 581/591 C11 lipid peroxidation probe to measure the level of lipid peroxidation in cells. The addition of rosiglitazone and oleic acid inhibited RSL3-induced lipid peroxidation, and oleic acid had a more significant effect on this level of lipid peroxidation ( Figure 3G,H). Therefore, the influence of the *NCOA6* gene on the expression of SCD1 is the main factor affecting RSL3-induced ferroptosis.

### *NCOA6* gene regulates gemcitabine resistance in pancreatic cancer

**Figure 4:**
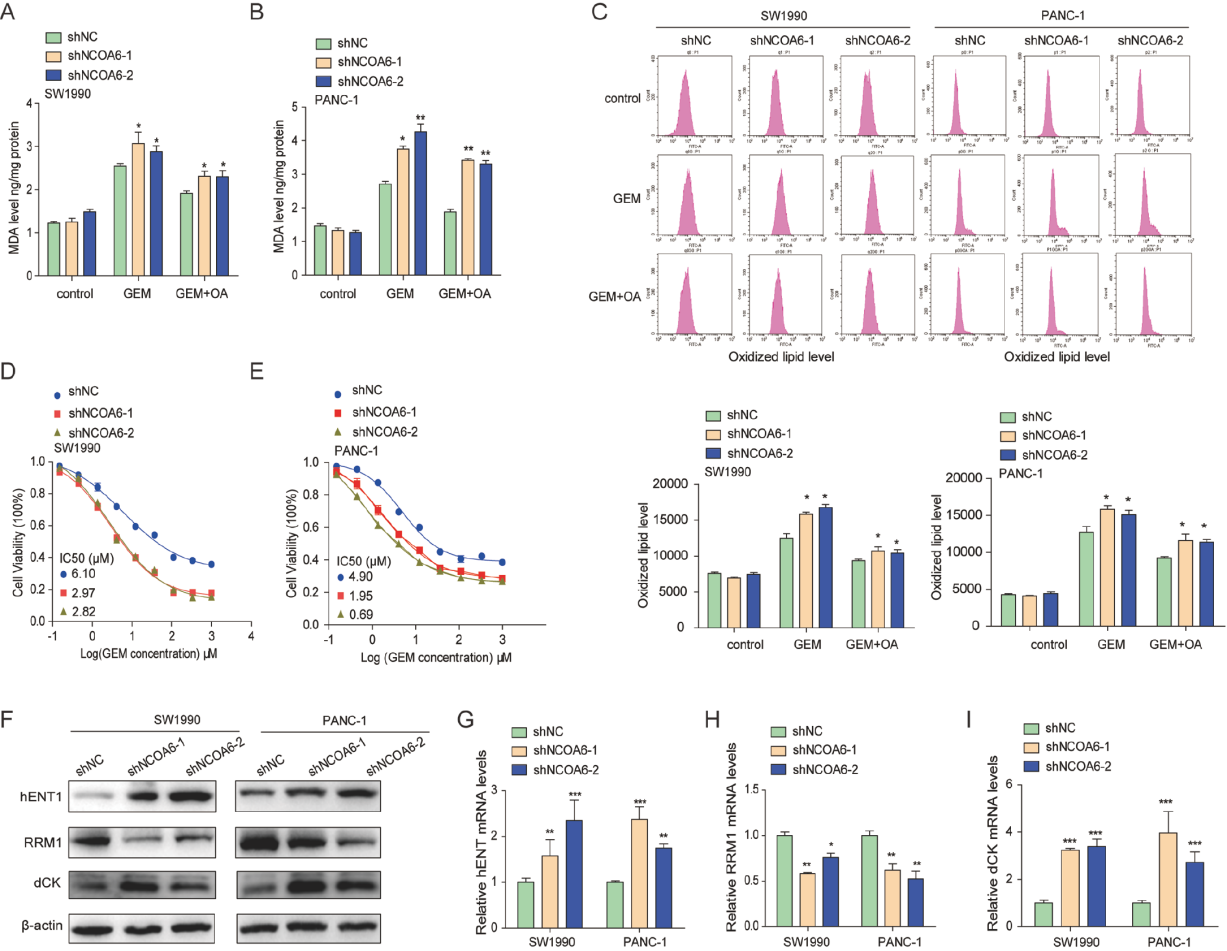
Figure 4 *NCOA6* gene regulates gemcitabine resistance in pancreatic cancer (A,B) The level of MDA in NCOA6-knockdown pancreatic cancer cell lines with or without OA pretreatment was detected. (C) Flow cytometry was used to measure the level of gemcitabine-induced lipid oxidation in OA-pretreated pancreatic cancer cells. (D,E) CCK8 assay was used to detect the dose-dependent toxicity of gemcitabine on pancreatic cancer cells with or without NCOA6 knockdown. (F) Western blot analysis of the protein expressions of hENT1, RRM1, and dCK. (G–I) qRT-PCR was used to detect the effects of NCOA6 knockdown on the mRNA levels of hENT1, RRM1, and dCK. *P < 0.05, **P < 0.01, ***P < 0.001.

Our study revealed that the NCOA6 gene regulates the sensitivity of pancreatic cancer cells to oxidative stress by influencing SCD1 and ACSL4. We found that gemcitabine induced lipid oxidation in pancreatic cancer cells. As shown in Figure 4A,B, after pancreatic cancer cells were stimulated with gemcitabine, the MDA content in the cells increased, and in *NCOA6*-knockdown cells, the increase in MDA content was more significant. After the addition of OA, the content of MDA decreased, indicating that the final product of SCD1, oleic acid, partially decreased the degree of lipid peroxidation caused by gemcitabine ( Figure 4A,B). The same phenomenon was found in the detection of lipid peroxidation levels in cells via the BODIPYTM 581/591 C11 lipid peroxidation probe ( Figure 4C). We further confirmed through CCK8 experiments that *NCOA6* knockdown increased the sensitivity of pancreatic cancer cells to gemcitabine. After stimulation with gemcitabine, the viability of *NCOA6*-knockdown cells was significantly lower than that of wild-type cells ( Figure 4D,E). NCOA6 affects the sensitivity of pancreatic cancer cells to gemcitabine by increasing lipid oxidation, but the mechanism might be multifaceted. To explore other mechanisms involved in the increased sensitivity of *NCOA6*-knockdown cells to gemcitabine, we detected the expressions of several genes related to gemcitabine resistance. Western blot analysis and qRT-PCR results confirmed that the expressions of hENT and dCK were upregulated and that the expression of RRM1 was downregulated in pancreatic cancer cells with *NCOA6* knockdown ( Figure 4F–I). Therefore, knockdown of the *NCOA6* gene can improve the sensitivity of pancreatic cancer cells to gemcitabine through various mechanisms.

### NCOA6 is positively correlated with SCD1 and negatively correlated with ACSL4 in clinical specimens

**Figure 5:**
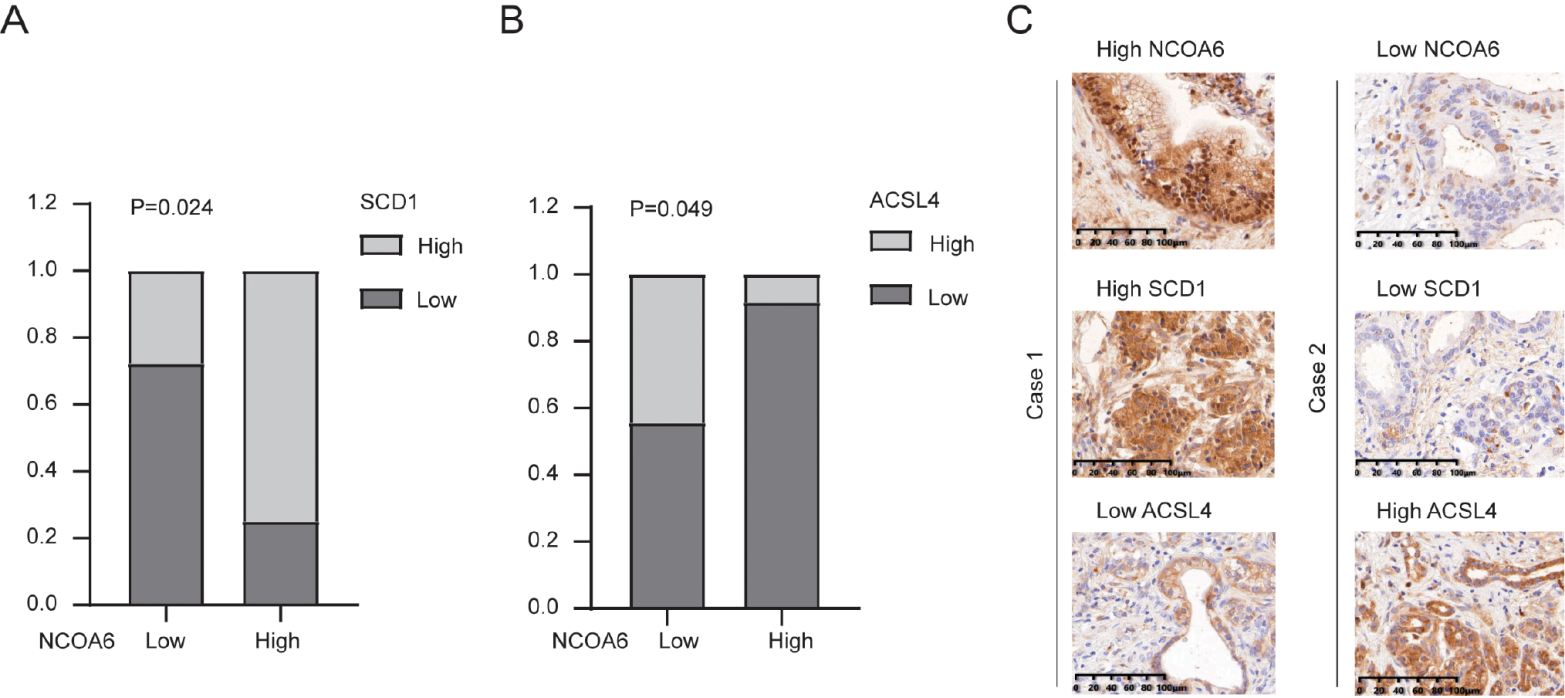
Figure 5 NCOA6 is positively correlated with SCD1 and negatively correlated with ACSL4 in clinical specimens (A,B) The relationships between SCD1 expression and NCOA6 and ACSL4 expressions were analyzed via Fisher’s exact probability test. (C) The expressions of NCOA6, ACSL4 and SCD1 in two typical cases.

To verify the expressions of NCOA6, SCD1 and ACSL4 in patient tissues, we randomly selected 30 patients with pancreatic cancer from our center, and immunohistochemical staining was performed on their paraffin-embedded tissues with corresponding antibodies. We multiplied the proportional scores of NCOA6, SCD1, and ACSL4 by the intensity score to calculate the IHC score. NCOA6, SCD1, and ACSL4 were further divided into high and low groups according to the IHC score. Statistical analysis was subsequently conducted on the relationships among NCOA6, SCD1, and ACSL4. The results revealed that NCOA6 expression was positively related to SCD1 expression in pancreatic cancer tissues and negatively related to ACSL4 expression ( Figure 5A,B). In addition, typical IHC images are shown in Figure 5C.

## Discussion

Cancer treatment has always been plagued by chemotherapy drug resistance. Inducing cell apoptosis is the most common form of anti-tumor effect of chemotherapy drugs. In addition to apoptosis, autophagy, necroptosis and ferroptosis are also forms of cell death, each with unique biological processes and pathophysiological characteristics. Ferroptosis is a non-apoptotic programmed cell death process characterized by the accumulation of lipid peroxidation.

Mutation or abnormal activation of the RAS superfamily promotes cancer progression. It is the most common mutant gene in human cancers, especially in pancreatic cancer, intestinal cancer and lung cancer [ 20, 21]. In 2003, erastin was found to have a selective lethal effect on cancer cells expressing RAS [22]. RSL3 has also been found to kill cancer cells with RAS mutations [ 22, 23]. Researchers have reported that erastin and RSL3 kill tumor cells in an iron-dependent manner rather than through cell apoptosis. In terms of mechanism, RSL3 induces ferroptosis by inhibiting GPX4, whereas erastin induces ferroptosis by inhibiting the Xc system [4].

NCOA6 is a co-activator that can bind to NRs [ 24– 26]. It is overexpressed in various cancers. Our research revealed that its expression level was greater in pancreatic cancer cell lines than in the normal pancreatic cell line H6C7. It is also overexpressed in the tumor tissues of pancreatic cancer patients. Research has shown that NCOA6 is related to the invasion and metastasis of tumor cells, but the relationship between NCOA6 and ferroptosis has not yet been reported. Our study revealed the role of NCOA6 in the ferroptosis of pancreatic cancer cells by knocking down *NCOA6* in pancreatic cancer cells and exploring the ferroptosis of these cells.

We found that the knockdown of *NCOA6* did not affect the level of lipid peroxidation in pancreatic cancer cells but could enhance RSL3-induced peroxidation. We found that NCOA6 can affect the expressions of SCD1 and ACSL4 in pancreatic cancer cells. *NCOA6* knockdown upregulated the expression of ACSL4 and downregulated the expression of SCD1. Ferroptosis is closely related to an imbalance in lipid metabolism, and polyunsaturated fatty acids (PUFAs) are highly sensitive to lipid peroxidation. PE is a key phospholipid associated with ferroptosis [27]. ACSL4 is involved in the biosynthesis and remodellng of PE, activates polyunsaturated fatty acids, and affects the transmembrane function of polyunsaturated fatty acids. The expression of ACSL4 increases the sensitivity of cells to ferroptosis-inducing compounds. SCD1-catalyzed production of monounsaturated fatty acids (MUFAs) can effectively inhibit ferroptosis by replacing PUFAs in the plasma membrane and reducing lipid ROS accumulation [28]. *NCOA6* knockdown in pancreatic cancer cells results in the downregulation of ACSL4 and the upregulation of SCD1, thus enhancing the lipid peroxidation caused by RSL3 in pancreatic cancer cells and increasing their sensitivity to ferroptosis. Sterol regulatory element binding protein 1 (SREBP1) is a well-known master regulatory factor involved in fat formation that can promote the growth and metastasis of cancer cells [ 29, 30]. It is a downstream effector of ACSL4, which regulates various lipid-generating enzymes, including SCD1, through the c-Myc/SREBP1 pathway. SCD1 is the target gene of SREBP1, and downregulation of SREBP1 can lead to a decrease in SCD1 expression [18]. Therefore, SREBP1-mediated adipogenesis is crucial for the ferroptosis process [17]. Our research confirmed that NCOA6 not only regulates the expressions of ACSL4 and SCD1 but also confirms the interaction between ACSL4 and SREBP1.

Ferroptosis has broad clinical application prospects, and it has been confirmed that drug-resistant tumor cells are sensitive to ferroptosis [31]. Inhibiting or activating ferroptosis can regulate the sensitivity of tumors to cisplatin [32]. In pancreatic cancer, ferroptosis regulators can enhance the killing effect of chemotherapy drugs on tumors [33]. Increasing evidence suggested that ferroptosis can serve as a potential target for treating chemotherapy-resistant tumors, as it can reverse resistance to chemotherapy drugs [34]. Our research revealed that knockdown of the *NCOA6* gene not only increased the sensitivity of pancreatic cancer cells to ferroptosis but also increased the sensitivity of tumor cells to gemcitabine. We found that gemcitabine can cause a certain degree of lipid peroxidation in pancreatic cancer cells, which may partially explain why *NCOA6* knockdown increases the sensitivity of pancreatic cancer cells to gemcitabine; that is, it increases the sensitivity of pancreatic cancer cells to gemcitabine-induced lipid peroxidation. We also explored the reasons for the increased sensitivity of *NCOA6* knockdown to gemcitabine from the perspective of the mechanism of gemcitabine resistance. *hENT*, *dCK* and *RRM1* are genes related to drug resistance in pancreatic cancer [ 35– 38]. We found that the knockdown of the *NCOA6* gene indeed regulated the expressions of these genes, which may explain why the knockdown of *NCOA6* can enhance the sensitivity of pancreatic cancer cells to gemcitabine.

The limitation of this study is that we did not further elucidate the specific mechanisms by which *NCOA6* regulates ACSL4 and SCD1, and further research is required to address this issue.

**Figure 6:**
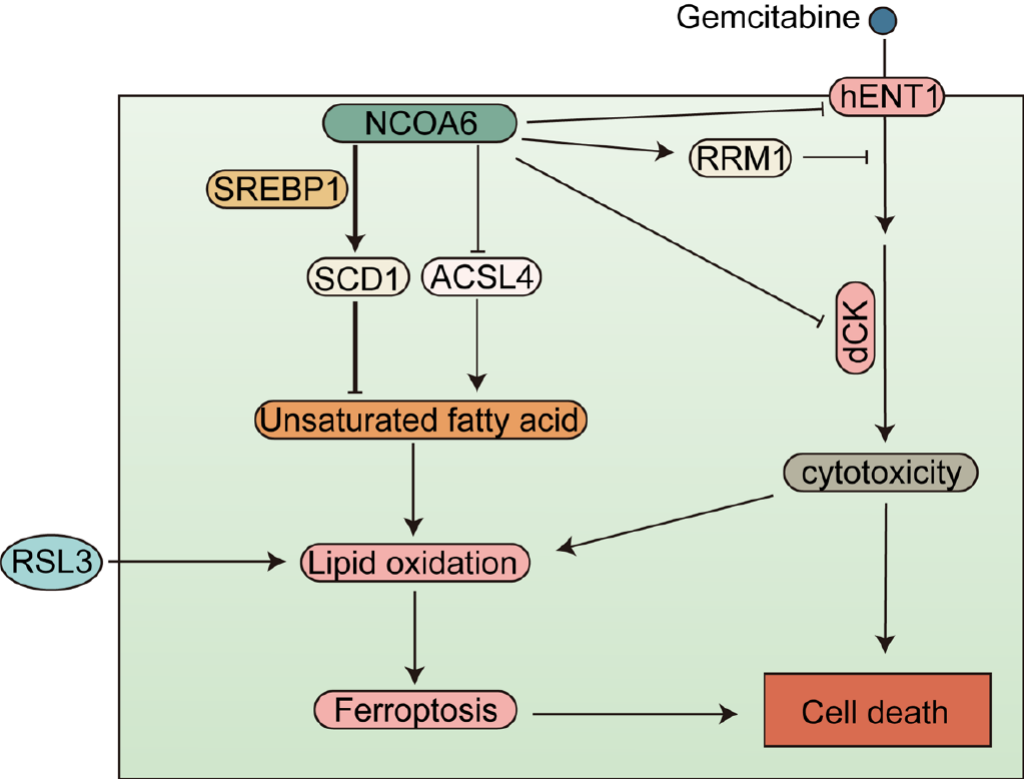
Figure 6 The graphical summary of this study The graphical summary describes how NCOA6 affects the ferroptosis-sensitive state of cells by regulating ACSL4 and SCD1, as well as the sensitivity of cells to gemcitabine by regulating hENT1, RRM1, and dCK, and ultimately regulates cell death.

In conclusion, this study revealed the role of the *NCOA6* gene in regulating RSL3-induced ferroptosis and gemcitabine resistance in pancreatic cancer cells and preliminarily explored the underlying mechanism ( Figure 6).
